# Phase Change Material Used for Masonry Joints to Reduce the Thermal Bridge Effect

**DOI:** 10.3390/ma12121895

**Published:** 2019-06-12

**Authors:** Wei Jiang, Dan Liu

**Affiliations:** 1 Key Laboratory of Advanced Civil Engineering Materials of Ministry of Education, Tongji University, Shanghai 201804, China; 2 School of Materials Science and Engineering, Tongji University, Shanghai 201804, China; 1730592@tongji.edn.cn

**Keywords:** phase change material, finite element analysis, thermal bridge, heat transfer coefficient, latent heat

## Abstract

In this paper, a numerical calculation and application analysis of composite phase change material masonry mortar applied to wall parts are performed during the research process. Instead of the conventional “sandwich” phase change material wall, our research group mainly uses phase change materials in the wall parts to build masonry joints to reduce the thermal bridge effect. The influence of masonry joints on the heat transfer of the wall is demonstrated. A quantitative description of the transient heat transfer coefficient is obtained to measure the heat preservation performance of the phase change material wall. Furthermore, the influence of different proportions of phase change materials on the wall heat transfer in different external environments is discussed, supplemented by the influence of the working range and sensitivity on the heat transfer. In summary, the use of phase change materials in the construction of masonry joints is a great innovation for conventional “sandwich” phase change material walls, optimizing the form, the thermal bridge effect and the heat preservation performance of wall parts. The quantitative description of the transient heat transfer coefficient expands the development of wall heat transfer theories. In addition, the conclusions are of great guiding significance for the structure and the phase change material’s blending proportion for the innovative heat preservation phase change material wall.

## 1. Introduction

### 1.1. Importance of Energy Conservation and Emissions Reduction

Saving energy, reducing emissions and having a low-carbon economy have become primary areas of focus across the globe, with building energy consumption in particular being a difficult problem to solve. Throughout the entire lifecycle of housing construction, 1/3 of the steel, 60–70% of the cement, 1/3 of the urban construction land, 1/3 of the urban water, and 40–50% of the energy are consumed, which has a large impact on energy, resources and the environment. Therefore, building energy consumption is an important area of ecological civilization construction [1]. Specifically, the heat storage and heat transfer of buildings are fundamental control links in building energy-saving technology. Currently, all countries regard the development of heat preservation materials as the main focus of building energy saving measures and are developing a series of heat preservation materials, such as foam polystyrene board and rock wool. In addition, the energy consumption of a building structure can be effectively reduced by the methods of composite wall design and the ecological function of vegetation. The development and popularization of the application of composite phase change materials (PCMs) in walls has also become an effective method of achieving energy savings. Therefore, the development of a new, green, light wall with heat storage and energy storage properties along with the promotion of new materials and technologies can also be effective for future buildings, which has laid a solid foundation for the development of low-carbon technology and a green economy.

### 1.2. Literature Review

Wall insulation technology is currently becoming increasingly important, and phase change materials have been extensively studied for their thermal storage potential in light-weight heat preservation walls. For example, Zhou studied a phase change material wall and reported that the energy savings can be as high as 40% compared to a similar building without PCMWs in the summer, and the period during which temperatures are maintained within the thermal comfort range can be improved by up to 7.2% [2]. The microencapsulated phase change materials (2.5%) were integrated into building walls by Liu, who discovered that the inner temperature of model house remained 2 K higher than without PCM during the test process [3]. A numerical study of PCMW used in a typical office building for two months that showed PCMW can averagely reduce the temperature of 9.22 K in the building operation time (7:00–18:00) in the summer [4]. Forzano [5] found that the energy saving percentage potential per cubic meter of phase change materials ranges from 1.9%/m^3^ to 18.8%/m^3^.

Until now, the encapsulation method, which mixes PCMs into the building materials, has mostly been used in a “sandwich” phase change material wall. A type of wallboard composed of new PCMs to improve thermal comfort and reduce energy consumption was demonstrated by Kuznik [6], who used CODYMUR software to model and optimize phase change material wallboard (PCMW) by changing the thickness of the heat preservation materials and PCMs. Finally, the optimum PCM thickness was calculated to be 1 cm for construction. Researchers [4] experimented with a new type of PCMW with a stable shape, which showed that the PCMW used in buildings may minimize the indoor temperature by 2.53 K. A three-layer “sandwich” insulation structure was newly proposed [7], which contains PCMWs on the outside and a traditional insulation material on the inside.

Currently, numerical calculation methods have been effectively applied to the vital part of energy saving analysis of wall envelope structure. Enthalpy methods [8] were applied to establish the phase change heat transfer model and were solved by the finite difference method. Furthermore, it was proposed that the enthalpy method was the most suitable [9] for solving the dynamic boundary of phase change heat transfer problems. A numerical study [10] of the phase change material wall was conducted, and the heat transfer process of the phase change material wall was analyzed. In addition, the thermophysical properties and melting process of a new energy storage system in some studies were numerically calculated [11]. The thermal performance of the phase change material wall and floor room was simulated by Zhou [12], and its application effect was analyzed from many aspects. A simplified dynamic model [13,14,15] of the building structure integrated with a double-deck SSPCM, which can provide an accurate and reliable thermodynamic prediction of the building structure integrated with double-deck SSPCMs, was proposed. Costanzo’s study [16], based on dynamic simulations of a typical office building, was carried out with EnergyPlus, with the aim of calculating the indoor operative temperature and the cooling load under thermostatic control.

Consequently, phase change material walls have large energy storage capacities and do not require indoor space, while also potentially reducing the weight and thickness of the wall. However, it can be observed that the use of phase change materials in wall research is currently limited to the “sandwich” PCM wall, which has a single form and insufficient thermal storage potential. Furthermore, so much PCMs is needed in the “sandwich” PCM wall that the cost is prohibitively expensive, so it cannot be applied widely. Therefore, in order to simultaneously reduce the thermal bridge effect and reduce the cost of the phase change material, the phase change material was added only in the masonry joint on the basis of the existing traditional masonry brick wall. In the attempt to model our new phase change material wall structure more precisely, Abaqus is a more reliable tool used to achieve instantaneous heat flux and instantaneous temperature value. A quantitative description of the transient heat transfer coefficient to measure the heat preservation performance of the phase change material wall was attempted.

In summary, the use of PCMs in the construction of masonry joints is a great innovation for conventional “sandwich” phase change material walls, optimizing the form, the thermal bridge effect, and the heat preservation performance of wall parts. In addition, the numerical calculation of unsteady heat transfer of wall parts can be expressed by the instantaneous heat transfer and instantaneous temperature value; the output value of the heat transfer can be abstracted into an unsteady heat transfer coefficient equation and verified, thereby simplifying judgement methods on the heat transfer performance of the phase change material wall.

### 1.3. Research Ideas in This Paper

Compared with the conventional “sandwich” phase change material wall, our research group mainly used phase change materials in the wall parts to build masonry joints to reduce the thermal bridge effect.

First of all, the influence caused by the existence of masonry joints on the heat transfer of the wall was demonstrated and a composite phase change material masonry mortar was used to replace the original common mortar to reduce the thermal bridge effect.

Secondly, a quantitative description of the transient heat transfer coefficient to measure the heat preservation performance of the phase change material wall was verified by comparing it with the results of the traditional theoretical equation.

Finally, the influence of different proportions of phase change materials on the wall heat transfer in different external environments was discussed, supplemented by the influence of the working range and sensitivity on the heat transfer.

## 2. Materials and Methods

The purpose of this paper is to study the transient heat transfer of the wall with the actual temperature change during one day, and to obtain a quantitative description of the transient heat transfer coefficient to measure the thermal storage performance of the phase change material wall, so as to compare the thermal storage performance of wall with different proportions of PCM used for the masonry joints. It is of great guiding significance for the structure and the phase change material’s proportion of the innovative heat preservation phase change material wall.

### 2.1. Introduction of Materials and the Wall Structures

A composite phase change thermal storage material is the main research object of this paper. This material can not only effectively overcome the shortcomings of single inorganic or organic phase change materials, but also improve the application effect of phase change materials and expand their application scope. In this paper, decanoic acid is taken as an example. The solid-phase temperature of decanoic acid is 31.5 °C, and the liquid-phase temperature is 38.5 °C. The complete working temperature range of decanoic acid is 31.5 °C < T < 38.5 °C, and its specific thermal parameters are given in Table 1. Furthermore, all materials of the studied PCM wall are listed in Table 1.

**Figure 1:**
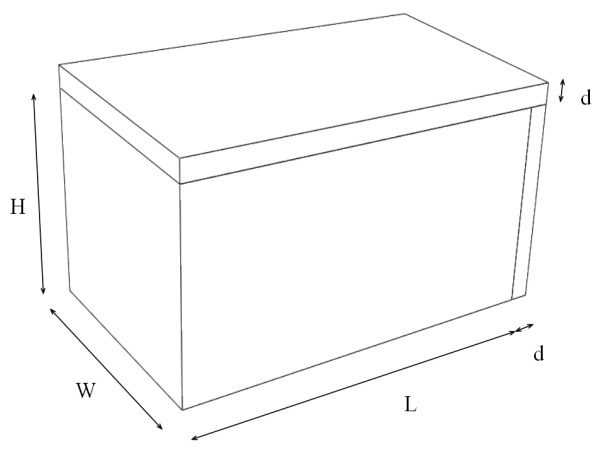
Schematic diagram of basic unit of block wall.

The research object of this study is the basic wall component unit composed of a standard wall block and standard mortar, as shown in Figure 1. The length, the width and the height of the block is LWH and the width of masonry joints is d. According to the size of standard wall blocks and the width of standard masonry joints, wall parts consisting of 390 mm × 240 mm × 190 mm blocks and 10 mm wide masonry joints were studied.

**Figure 2:**
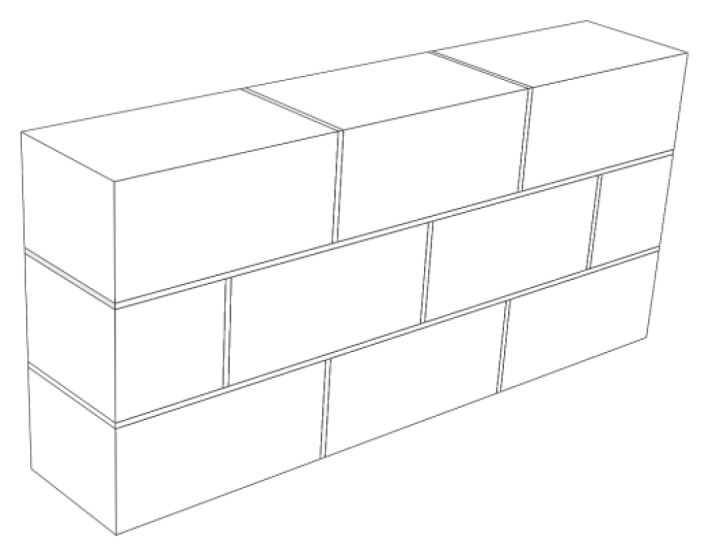
Schematic diagram of the three-layer staggered-joint wall.

Using Abaqus finite element analysis software to simulate the heat transfer of wall parts under steady state can visually show the heat transfer of different materials in different areas of wall parts, indirectly indicating their heat preservation and storage function. To better reflect the heat transfer situation in different areas to obtain more accurate and qualitative analysis results, the research object was chosen to be a three-layer staggered-joint wall. The main body of the studied wall consists of nine blocks, the total volume of which is equal to the block and masonry joints of the unit. There are two horizontal masonry joints between the three-layer walls, and the longitudinal masonry joints are from top to bottom. The three layers are two, three, and two blocks, respectively, as shown in Figure 2.

Assuming that the joints between the blocks and mortar are homogeneous materials, basic calculation parameters such as the density, specific heat, thermal conductivity, and latent heat of each material should be provided before the heat transfer simulation in the wall model. In this study, the materials’ parameters of mortar, slag cotton brick, decanoic acid, concrete, lightweight insulating brick were used for comparisons and compounding calculations. All materials’ general thermal parameters are shown in Table 1.

### 2.2. Traditional Theoretical Equation for the Wall Heat Transfer Coefficient

The main heat transfer direction of the wall is along the thickness of the wall and inside and outside the wall. In the direction of heat transfer, the thermal resistance of the block and masonry joints is parallel, the basic unit of the wall is in series with the internal and external heat transfer resistances, and the heat transfer coefficient is equal to the reciprocal of the total heat resistance of the wall. Therefore, the theoretical equation for calculating the heat transfer coefficient *K* is as follows [17]:

(1) $$K=\frac{1}{R}=\frac{1}{\frac{1}{h_{\mathrm{out}}}+\frac{LH+Hd+(L+H)d}{\frac{LH}{W/\lambda_{B}}+\frac{Hd}{W/\lambda_{\mathrm{MJ}}}+\frac{(L+H)d}{W/\lambda_{\mathrm{MJ}}}}+\frac{1}{h_{\mathrm{in}}}}$$

In Equation (1), the internal (*h*_in_) surface coefficient of heat transfer and external (*h*_out_) surface coefficient of heat transfer of the walls are taken as the commonly used empirical values of 19 (W·m^−2^·°C^−1^) and 8.7 (W·m^−2^·°C^−1^), respectively. $\lambda_{B}$ and $\lambda_{\mathrm{MJ}}$ are the thermal conductivity of the blocks and masonry joints, respectively. *L*, *W* and *H* are the length, width, and height of the wall, respectively. *d* is the width of the masonry joints.

A model of two blocks with masonry joint shown in Figure 1 was established. The main materials of wall parts were placed into three groups, as shown in the second column of Table 2, and the material properties shown in Table 1 were assigned. Finally, the theoretical heat transfer coefficients of these groups were calculated by Equation (1) and listed in the third column of Table 2.

### 2.3. The Derived Equation for the Wall Steady-State Heat Transfer Coefficient

It can be seen in Equation (1) that, although the theoretical equation is accurate for the study on the heat transfer of wall parts, the calculation process of the total thermal resistance is extremely complicated when dealing with a more complex heat transfer of the wall parts, such as filled bricks, for that the inner part of the wall is divided into a multi-area and multi-material complex grid system. Therefore, a new method by numerical calculations is necessary and a new equation is essential for Abaqus calculation.

A wall structure the same as the model in Section 2.2 was established, and the material properties shown in Table 1 were assigned. The steady-state temperature boundary conditions were set as follows:

(2) $$\left\{ \begin{array}{l} T_{\mathrm{out}}=T_{1}\\ T_{\mathrm{in}}=T_{2} \end{array}\right.$$

- In Equation (2), $T_{\mathrm{out}}$ and $T_{\mathrm{in}}$ are the temperature boundary conditions of the outer and inner surfaces of the wall, whose values are 40 °C and 30 °C, respectively. The initial temperature of the wall parts is the same as the inner surface temperature. In the process of a numerical calculation of wall parts using a finite element analysis, the research object is divided into a large number of micro-elements, and the entire heat transfer situation can be reflected by the interference superposition of the heat transfer situation of countless elements that are in steady state. Therefore, the derived heat transfer coefficient equation can be deduced reversibly by using the element mean given in the field output of the finite element analysis software as follows:
  (3) $$K_{d}=\frac{2Q_{\mathrm{AHF}}\cdot n_{W}\cdot n_{H}}{W\cdot H\cdot \Delta T}.$$
- In Equation (3), the average heat transfer (*Q*_AHF_) of a single grid integral point under steady state of the wall is Watt, which is directly given by the output file of the finite element analysis software field; $n_{W}$ and $n_{H}$ are the number of grids in the direction of the thickness and height extension paths of the wall, respectively; and ΔT is the temperature range between the inner and outer surface of the wall in steady state.

**Figure 3:**
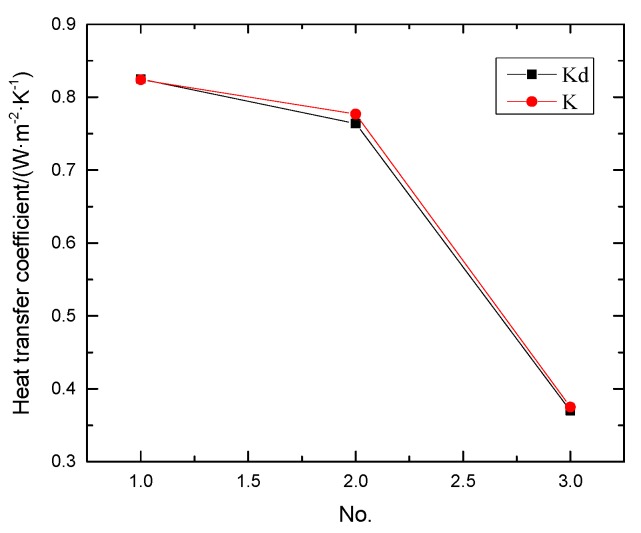
Results comparison of theoretical calculation method and the derived calculation method.

To verify Equation (3), three models were built with the same parameters in Section 2.2 by the numerical simulation and the average heat transfer (*Q*_AHF_) was achieved in the analysis software. Finally, Equation (3) was used to calculate these groups’ heat transfer coefficient *K*_d_. The results were listed in the last column of Table 2, while the results of Section 2.1 are listed in the third column of Table 2. In other words, the results of theoretical heat transfer coefficient Equation (1) and derived heat transfer coefficient Equation (3) are both shown in Table 2. A results comparison of the theoretical calculation method and the derived calculation method is shown in Figure 3.

According to the data from the third and last column in Table 2 and Figure 3, the theoretical heat transfer coefficient K, is approximately equal to the value of the derived heat transfer coefficient Kd when the properties of different composite materials are given by the same model. The accuracy of the derivative calculation method is confirmed, and the analysis of the derivative calculation method in later research is also guaranteed. Therefore, the feasibility of the following calculation is confirmed.

**Figure 4:**
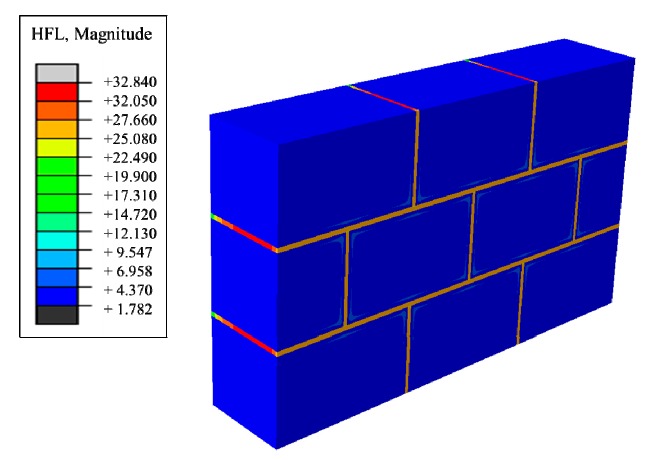
Heat flux nephograms of steady heat transfer.

Next, aiming at improving the heat preservation and storage function more efficiently, effects of the existence of masonry joints on the thermal performance of traditional masonry wall was studied. A wall structure of Figure 2, introduced in Section 2.1, was built and, from the heat flux nephogram in Figure 4, it can be seen that the heat flux region adjacent to the block and the masonry joints produces a gradual change region, indicating that the heat preservation performance of the block area adjacent to the masonry joints is weaker than that of the central area of the block due to the influence of the masonry joints. In summary, the existence of masonry joints weakens the insulation performance of the entire wall, which greatly reduces the insulation performance.

**Figure 5:**
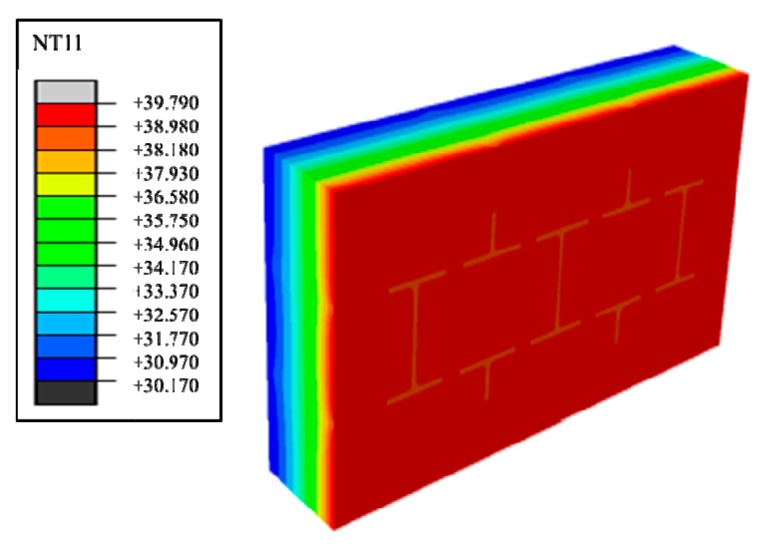
Steady heat transfer heat flux nephogram (containing decanoic acid).

**Figure 6:**
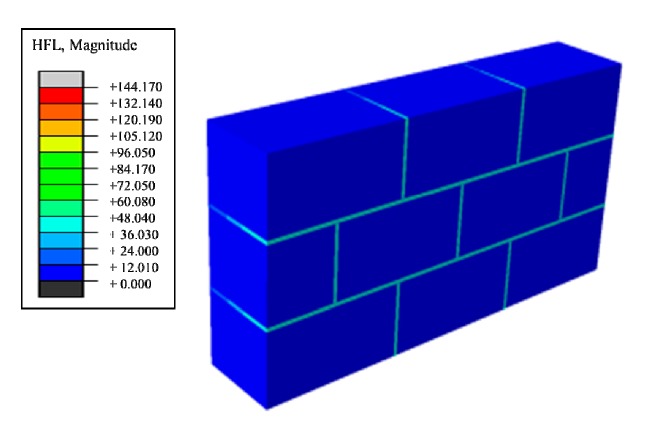
Steady heat transfer temperature nephogram (containing decanoic acid).

**Figure 7:**
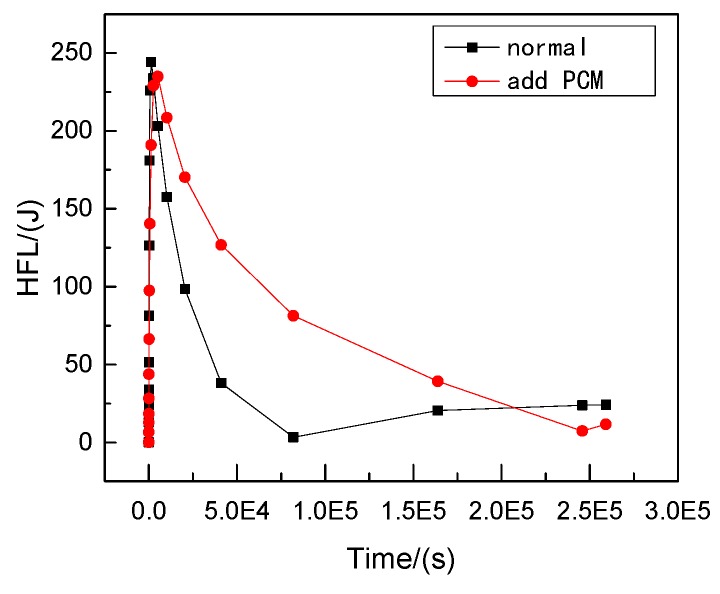
Steady-state heat transfer heat flux characteristic curve (containing decanoic acid).

**Figure 8:**
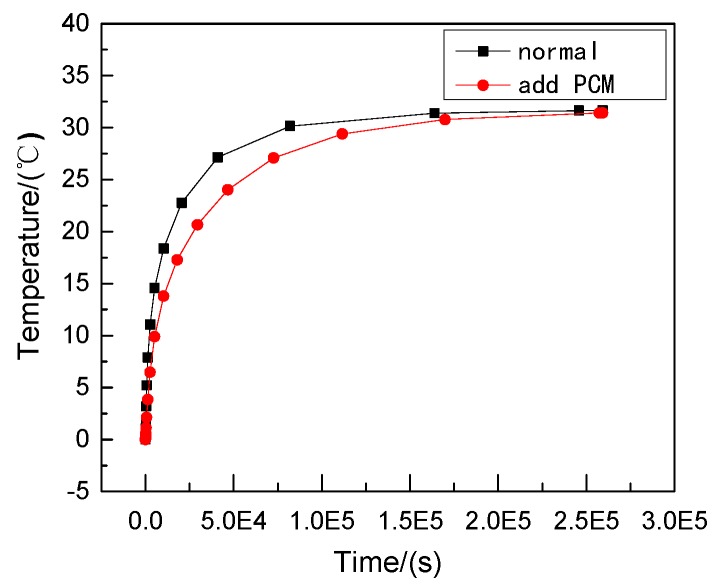
Steady-state heat transfer temperature characteristic curve (containing decanoic acid).

Therefore, phase change materials, such as decanoic acid, are added into masonry joints, and a composite phase change masonry mortar layer is used to replace the original common mortar layer. It is assumed that in composite phase change masonry mortar the benchmark incorporation mass percentage of decanoic acid is 3.5%. Assuming that the other conditions remain unchanged in Section 2.1, the temperature and heat flux nephograms at the same time as Figure 5 and Figure 6 are obtained. The heat flux and temperature characteristic curves at the same position (the center of masonry joints) are shown in Figure 7 and Figure 8, respectively.

It can be observed that, when the external wall’s ambient temperature and other conditions are the same, under the condition of steady heat transfer and with an increase in time, the masonry joint temperature with a 3.5% phase change material in the inner wall is always lower than that of the ordinary masonry joints, and the change in the maximum heat flux value of the former is less than that of the latter; that is, the heat transfer effect is more stable. Furthermore, the faster the heat flux is transmitted, the more heat flux is used to change the state of the phase change material, which leads to a reduction in the internal energy of the wall itself, and the temperature becomes lower.

In conclusion, the composite phase change material masonry mortar can reduce the decline in the overall heat preservation performance of the wall to a certain extent compared with ordinary mortar. This phenomenon indirectly proves that the heat preservation performance of the phase change material used for masonry joints to reduce the thermal bridge effect is better, so the derived equation for transient heat transfer coefficient in the PCM wall was demonstrated to quantitatively describe and compare the thermal performance more clearly in the following studies.

### 2.4. The Derived Equation for Transient Heat Transfer Coefficient in the PCM Wall

Although the heat transfer coefficient can determine the thermal conductivity and heat preservation performance of the wall under steady-state heat transfer, there is no steady-state heat transfer in the actual service environment. Therefore, in order to judge the thermal conductivity and heat preservation performance of wall parts more effectively in engineering applications, the numerical calculation method of thermal conductivity under transient heat transfer is introduced as follows:

(4) $$K_{m}=\frac{Q}{\Delta T\cdot \Delta t}.$$

**Figure 9:**
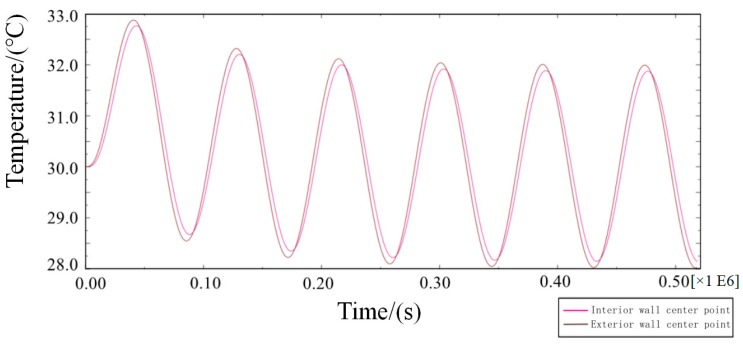
Six-cycle transient heat transfer characteristic point temperature curve.

**Figure 10:**
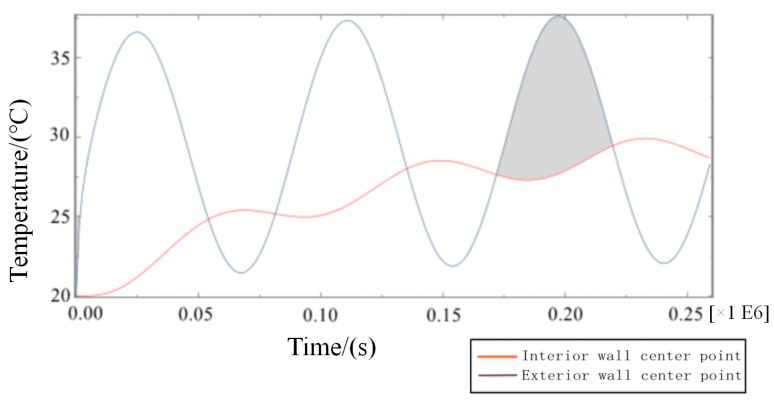
Transient heat transfer characteristic curve of internal and external wall temperature (high temperature).

- In Equation (4), Q is the total heat flux from the outer wall to the inner wall in a single period after the transient heat transfer temperature waveform changes stably, J/m^2^; Δt is the calculating effective time period, s.
  On the basis of model 2, the transient temperature boundary conditions are set as follows:
  (5) $$T_{\mathrm{out}}=10\sin\left(\frac{2\pi }{86400}t\right)+30.$$
- In Equation (5), the amplitude of the periodic function A = 10, the intercept C = 30, the integral refers to the ambient temperature of the external wall as a reference, and 10 °C is the up and down vibration amplitude fluctuation. Additionally, the initial phase is zero, referring to when t = 86,400 s, and the vibration completes a cycle to simulate the actual service time state from 6 a.m. to 6 a.m. the next day. To determine a reasonable cycle period in the study, the characteristic temperature curve is obtained by numerical calculation for the total step size of six days, as shown in Figure 9.
- From Figure 9, the following waveforms are almost unchanged from the third waveform, that is, when the cycle period is ≥3 days, the fluctuation range of temperature inside the wall tends to be stable because the true value of temperature cycle in the actual environment is ≤3 days. The maximum temperature difference in the process of heat transfer in the wall direction is the duration of the heat transfer process from the outer wall to the inner wall in a single cycle after the transient heat transfer temperature waveform changes stably, as shown in the shadowed area of the third cycle of the curve of the temperature change between the feature points of the outer wall and the feature points (the center of masonry joints) of the inner wall in Figure 10.

The transient heat transfer of wall parts with phase change materials (PCMs) in masonry joints is studied. In the process of heat transfer from the outer wall to the inner wall, PCMs store the heat, which should be transferred to the inner wall in the form of latent heat. Therefore, when using Equation (4) to calculate the transient heat transfer coefficient, it is necessary to make the following amendments:

(6) $${K_{m}}^{\prime }=\frac{Q-Q^{\prime }}{\Delta T\cdot \Delta t}.$$

In Equation (6), the effect of latent heat of the composite phase change material mortar($Q^{\prime }$) is taken into account.

**Figure 11:**
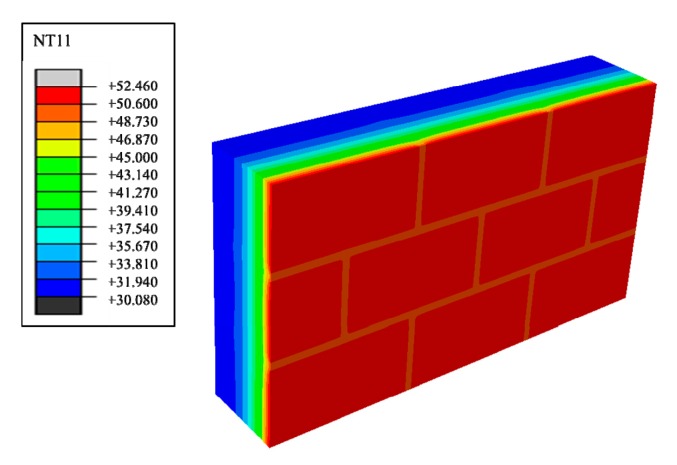
Transient heat transfer temperature peak nephogram.

**Figure 12:**
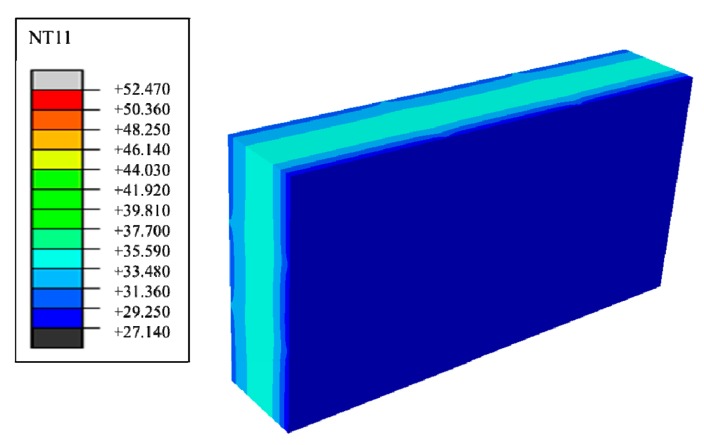
Transient heat transfer temperature low peak nephogram.

**Figure 13:**
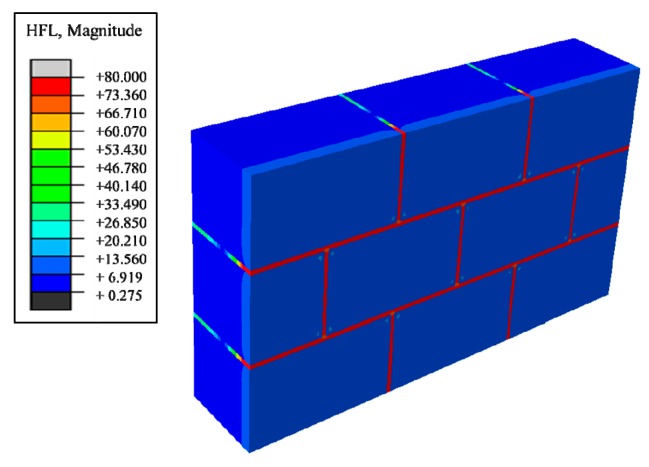
Transient heat transfer heat flux peak nephogram.

**Figure 14:**
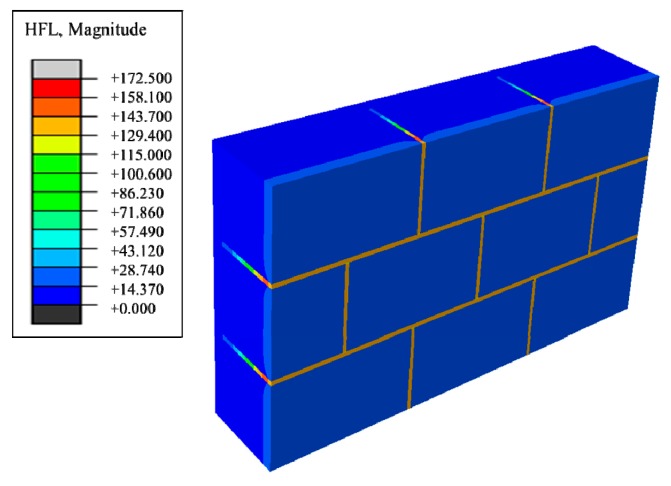
Transient heat transfer heat flux low peak nephogram.

**Figure 15:**
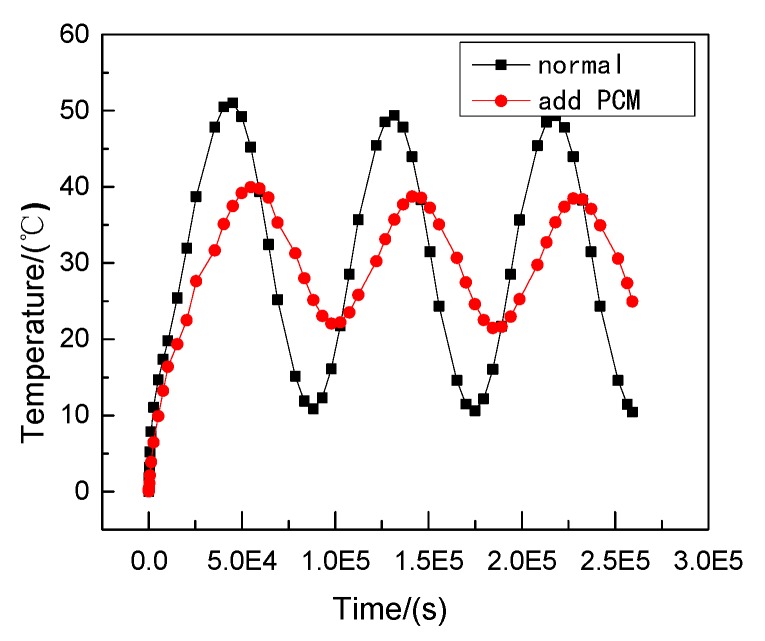
Transient heat transfer temperature characteristic curve.

**Figure 16:**
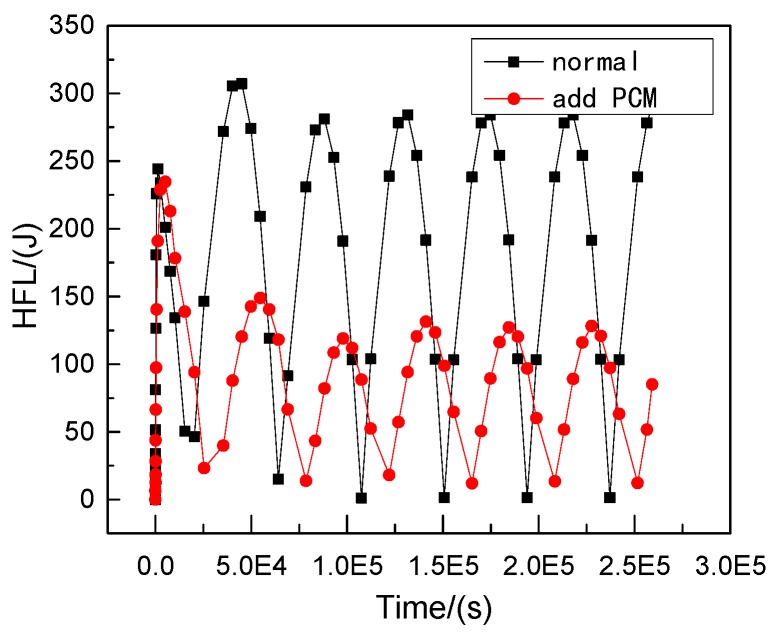
Transient heat transfer heat flux characteristic curve.

Suppose that the calculating effective time period of wall parts in this study is three days, which is 0 s < t < 259,200 s, under the condition of a regularly changing temperature. Phase change materials such as decanoic acid are added into masonry joints, and the composite phase change masonry mortar layer is used to replace the original common mortar layer. It is assumed that in the composite phase change masonry mortar the benchmark proportion of decanoic acid is 3.5%. Therefore, the phase change material wall of Figure 2 was numerically calculated in three days with the basic parameters introduced in Section 2.1 and Section 2.3, the value of Q and $Q^{\prime }$ in Equation (6) is obtained by summing the unit heat flux of each time in a single period after the transient heat transfer temperature waveform changes stably from the outer wall to the inner wall, which is conveniently achieved in the field output text of the Abaqus finite element analysis software. Finally, the temperature and heat flux nephograms can be obtained, and the characteristic curves can be drawn as shown from Figure 11, Figure 12, Figure 13, Figure 14, Figure 15 and Figure 16.

It is observed from Figure 11, Figure 12, Figure 13, Figure 14, Figure 15 and Figure 16 that when the ambient temperature of the exterior wall is the same, the peak value of the temperature and heat flux waveform of masonry joints mixed with 3.5% phase change materials in the interior wall is always lower than that of common masonry joints, while the low peak value of the fluctuation temperature is higher than that of common masonry joints, and the heat flux value of the former is the highest. It is determined that the amplitude of the composite phase change material masonry mortar wall part is lower than that of the ordinary masonry mortar wall part on the temperature curve of masonry joints at the same distance from the outer wall, and a certain time postposition occurs. In conclusion, the composite phase change masonry mortar can also improve and modify the overall heat preservation performance of wall parts during the actual service environment.

Taking the transient heat transfer model of the PCM mortar wall in Section 2.1 as an example, the modified heat transfer coefficient can be calculated by Equation (4):

$$K_{m}=\frac{Q}{\Delta T\cdot \Delta t}=\frac{255145.21(J\cdot m^{-2})}{15\;{}^{{}^{\circ}}C\cdot 360000\;s}=0.4725(W\cdot m^{-2}\cdot {}^{{}^{\circ}}C^{-1}).$$

Compared with the corresponding steady-state heat transfer, the heat transfer coefficient (K = 0.322 W·m^−2^·°C^−1^) is within the allowable error range. Therefore, Equation (4) can be used as a reference heat transfer coefficient to determine the transient heat transfer performance on the basis of a numerical calculation.

Taking the transient heat transfer model of the PCM mortar wall in Section 2.1 as an example, the modified heat transfer coefficient can be calculated by Equation (6):

$${K_{m}}^{\prime }=\frac{Q-Q^{\prime }}{\Delta T\cdot \Delta t}=\frac{246735.02(J\cdot m^{-2})-5000.00(J\cdot m^{-2})}{15\;{}^{{}^{\circ}}C\cdot 360000\;s}=0.4477(W\cdot m^{-2}\cdot {}^{{}^{\circ}}C^{-1}).$$

Compared with the corresponding instantaneous heat transfer coefficient (${K_{m}}^{\prime }$) of an ordinary mortar wall, its numerical value is slightly decreased, which is consistent with the conclusion obtained in Section 2.3. Therefore, on the basis of a numerical calculation, Equation (6) can be used as a reference to determine the transient heat transfer and heat transfer performance of wall parts containing phase change materials.

## 3. Results

The effects of phase change materials on the transient heat transfer of wall components under different external environments (high/low temperature) were studied by taking the proportion of phase change materials in the mortar as a variable.

### 3.1. High Temperature Phase Change Material’s Proportion

The phase change material as an admixture should be guaranteed to be less than 5% of the theoretical value in mortar. Now, in a high temperature environment, decanoic acid is taken as an example. Taking the model in Figure 2 as the research object, seven proportion of PCM between 2% and 5% are taken for the finite element analysis and calculation, and the reference heat transfer coefficient of the transient heat transfer is calculated and revised according to Equation (6). The set period of calculation is 3D, and the external wall temperature setting is shown in Equation (5). The result is shown in Table 3.

**Figure 17:**
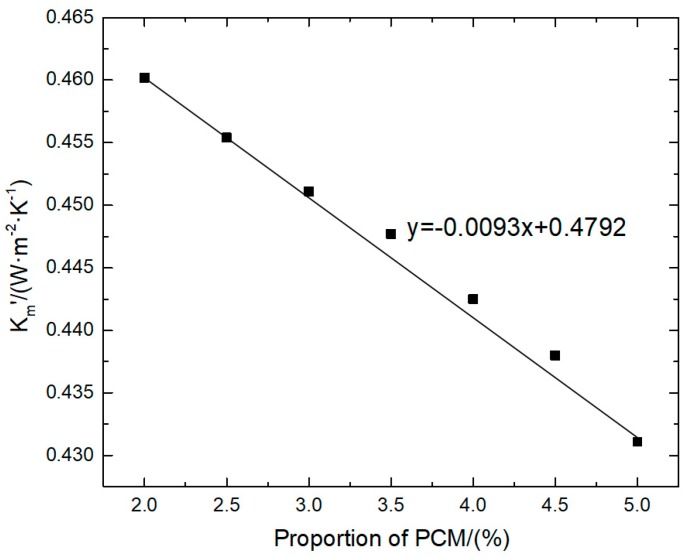
Heat transfer coefficient-proportion of PCM diagram.

According to the data in Table 3, the latent heat of the composite phase change mortar increases with an increase in the decanoic acid’s proportion, and the modified heat transfer coefficient decreases with an increase in the decanoic acid’s proportion. It can be seen that in the theoretical range of allowable admixture, that is, when the admixture is less than or equal to 5%, the larger the admixture of the phase change materials, the stronger the heat preservation performance of the corresponding wall parts. After drawing the scatter plot, the relative line of the trend line can be obtained, as shown in Figure 17.

According to the relationship between the value ${K_{m}}^{\prime }$ and the proportion of decanoic acid given in Figure 17, it can be determined that the fitting trend line is approximately a straight line, and the analytical equation of the straight line is as follows:

(7) y=−0.0093x+0.4792.

Equation (7) reflects the linear correlation between the correction coefficient of the transient heat transfer and the decanoic acid proportion. This correlation is referred to as the characteristic curve of the proportion of decanoic acid - heat transfer coefficient. When the proportion of the phase change material is 0%, the heat transfer coefficient of Equation (6) is 0.4792 (W·m^−2^·°C^−1^), which is approximately equal to the calculated value of the transient heat transfer coefficient in Section 2.4. When the proportion of the phase change material is 25%, the corresponding heat transfer coefficient is 0.2467 (W·m^−2^·°C^−1^), and it can meet the needs of high heat preservation and low thermal conductivity of walls in most special projects. Therefore, it can be determined that the definition domain of additive *x* in Equation (6) is 0 < *x* < 25.

In the application project of composite phase change masonry mortar wall parts with decanoic acid as the phase change material, the theoretical value of the decanoic acid’s proportion can be given according to the characteristic curve of the decanoic acid’s proportion-heat transfer coefficient, which can meet the requirements of the heat transfer coefficient of wall parts in engineering. It can also simplify the service while meeting the requirements of heat preservation performance of the wall parts. The related test of the engineering application can reduce cost, shorten the construction period and improve the overall construction benefit.

### 3.2. Low Temperature Phase Change Material’s Proportion

**Figure 18:**
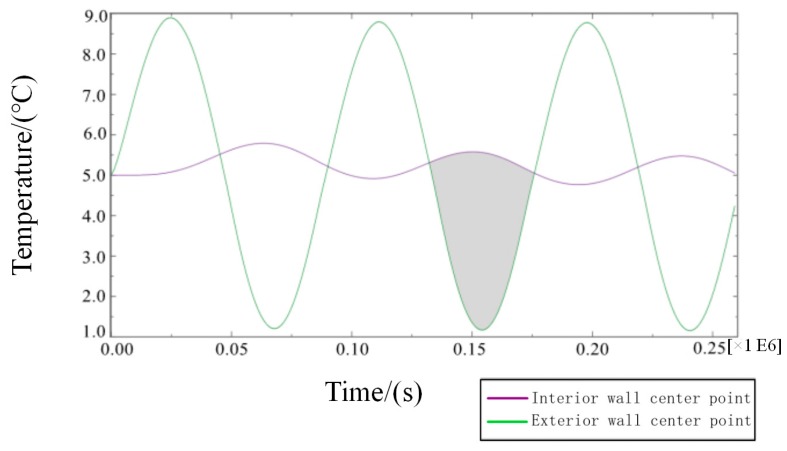
Temperature change curve of the internal and external walls of the transient heat transfer (low temperature).

Compared with the building heat preservation requirements under a high temperature, the low temperature state needs to minimize the loss of heat in the internal environment caused by wall heat transfer. Therefore, when calculating the transient correction heat transfer coefficient, the time and temperature range should be indicated in Figure 18.

Taking a cryogenic phase change material as an example, its latent heat is 50,000 J/kg, and the temperature range of the phase change is 4.5 °C < T < 8.5 °C. Similarly, the model of Figure 2 is taken as the research object. Based on the conclusions of Section 3.1, five proportions of 5% to 25% are selected for finite element numerical analysis and calculation, and the reference heat transfer coefficient of the transient heat transfer is calculated and repaired according to Equation (6). The calculation period is set to three days, and the temperature of the external wall is set to a fluctuation function with a fluctuation of 5 °C as the reference line and a fluctuation of 5 °C as the fluctuation function with the period of 24 h, as shown in Equation (8). The calculation results are shown in Table 4.

(8) $$T_{\mathrm{out}}=5\sin\left(\frac{2\pi }{86400}t\right)+5$$

**Figure 19:**
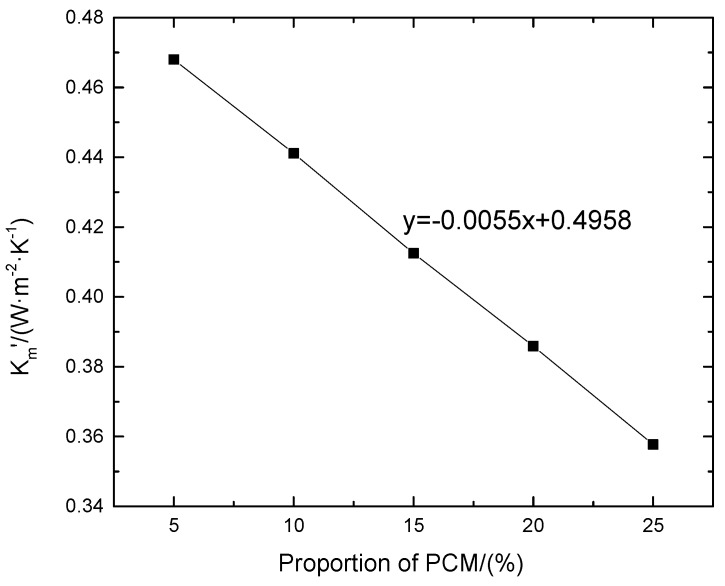
Heat transfer coefficient—phase diagram of the low temperature phase change material.

According to the data, the latent heat of the composite phase change mortar increases with an increase in the proportion of the cryogenic phase change material, and the modified heat transfer coefficient of the research object decreases with an increase in the proportion of the cryogenic phase change material. It can be seen that, in the same theoretical range as the high temperature phase change materials, that is, when the proportion of phase change materials is ≤25%, the larger the proportion of phase change materials is, the stronger the heat preservation performance of corresponding wall parts is. After drawing the scatter plot, the relative shape of the trend line can be obtained as shown in Figure 19.

According to the relationship between the value given in Figure 19 and the proportion of low temperature phase change materials, it can be determined that the fitting trend line is approximately a straight line, and the analytical equation of the straight line is as follows:

(9) y=−0.0055x+0.4958.

- Equation (9) reflects the linear correlation between the correction coefficient of the transient heat transfer and the proportion of a cryogenic phase change material. This equation is referred to as the characteristic curve of the proportion of a cryogenic phase change material-heat transfer coefficient. The definition domain of the proportion *x* is (0 < *x* < 25), and it can be determined from the figure that when the proportion of decanoic acid is 0%, the intercept obtained is 0.4958 (W·m^−2^·°C^−1^) when there is no phase change material, which is slightly different from the intercept of the characteristic curve of the decanoic acid’s proportion-heat transfer coefficient, and the latter is closer to the transient heat transfer coefficient. It can be seen that the correction of the transient heat transfer coefficient of wall parts containing phase change materials at a high temperature is more accurate than that of wall parts containing phase change materials at a low temperature.

From all of the numerical calculations and analysis in Section 3, it can be seen that the phase change materials used in composite phase change masonry mortar are different and the characteristic curves of the mixing quantity and heat transfer coefficient are different for models with definite specifications and properties of materials. Therefore, it is necessary to determine the characteristic curves of the mixing quantity and heat transfer coefficient according to engineering requirements in the process of practical engineering application. The required heat transfer coefficient of wall parts is within the range of the characteristic curve of the phase change material, so the type of phase change material and its corresponding proportion can be determined.

## 4. Discussion

The working temperature range of phase change materials is determined by the properties of the phase change materials themselves. Taking decanoic acid as an example, the solid-phase temperature and liquid-phase temperature of decanoic acid are 31.5 °C and 38.5 °C, respectively, and the complete working temperature range of decanoic acid is 31.5 °C < T < 38.5 °C. Figure 2 is taken as the research object. The composite phase change masonry mortar with 3.5% decanoic acid is used. Different temperature schemes of inner and outer walls are set as in Section 2 for a finite element analysis and calculation. The results are shown in Table 5.

It can be seen from the data in the table that, regardless of what the actual working temperature range is, when the working temperature range of the phase change materials studied becomes larger during the complete working temperature range, the time taken for the wall parts to stabilize will be longer, and the temperature of the wall and the masonry joints will be lower after stabilization. The larger the actual working temperature range of the obtained phase change material is, the better the insulation performance of the corresponding wall parts is.

## 5. Conclusions

The existence of masonry joints in the wall greatly reduces the heat preservation and storage function of the wall parts, making them the weakest link in the wall heat transfer. In this paper, a numerical calculation of the steady-state heat transfer and the transient heat transfer of wall parts are performed, and the feasibility of replacing common mortar with composite phase change masonry mortar in actual service environment is demonstrated to reduce the thermal bridge effect. The main research results are as follows:

- The transient heat transfer coefficient has been developed that can be used to characterize the heat preservation performance of wall parts under transient heat transfer. After adding phase change materials, such as decanoic acid, the correction method of the value is determined, and the transient correction heat transfer coefficient is obtained.
- The larger the temperature range of the phase change material is as a sub-range of the environmental temperature range, the better the effect of the phase change material is, and the range of the phase change material is unaffected by its location in the temperature range.
- The larger the mass fraction of the phase change material is in the composite phase change mortar, the more the heat preservation performance of the wall parts is improved, and the linear relationship is satisfied.

## Figures and Tables

**Table 1:** Thermal parameters of phase change materials.

Material	Density/(kg·m^−3^)	Thermal Conductivity/(W·m^−1^·°C^−1^)	Latent Heat/(J·kg^−1^)	Specific Heat/(J·kg^−1^·°C^−1^)
Decanoic acid	880	0.20	5000	/
Mortar	2000	0.87	/	820
Slag brick	400	0.07	/	800
Concrete	2500	1.74	/	920
Lightweight insulating brick	800	0.26	/	840

**Table 2:** Results of theoretical calculation method and the derived calculation method.

No.	Material	*K*/(W·m^−2^·°C^−1^)	*Q*_AHF_/(W)	*K*_d_/(W·m^−2^·°C^−1^)
1	Mortar, lightweight insulating brick and concrete	0.824	0.041	0.825
2	Mortar, concrete and slag brick	0.777	0.038	0.764
3	Mortar, lightweight insulating brick and slag brick	0.375	0.018	0.370

**Table 3:** Calculation results of different decanoic acid proportions.

Proportion/(%)	*Q*/(J·m^−2^)	Compound Latent Heat/(J·kg^−1^)	*K*_m_′/(W·m^−2^·°C^−1^)
2	251,361.77	2857	0.4602
2.5	249,415.34	3571	0.4554
3	247,872.41	4286	0.4511
3.5	246,735.02	5000	0.4477
4	244,682.50	5714	0.4425
4.5	242,945.82	6429	0.4380
5	239,951.68	7143	0.4311

**Table 4:** Statistical results of different low temperature phase change materials.

Proportion/(%)	*Q*/(J·m^−2^)	Compound Latent Heat/(J·kg^−1^)	$T_{\mathrm{out}}$/(W·m^−2^·°C^−1^)
5	86,740.399	2500	0.4680
10	84,398.755	5000	0.4411
15	81,750.851	7500	0.4125
20	79,462.629	10,000	0.3859
25	76,886.171	12,500	0.3577

**Table 5:** Statistical results of different decanoic acid working intervals.

$T_{\mathrm{in}}$/(°C)	$T_{\mathrm{out}}$/(°C)	ΔT/(°C)	Δt/(s)	Stabilized Wall Centre Temperature/(°C)	Stabilized Masonry Joints Centre Temperature/(°C)
30	40	7	133,200	34.913	34.921
33	40	5.5	128,400	34.939	34.959
30	37	5.5	128,400	34.942	34.960
35	40	3.5	124,800	34.975	35.002
30	35	3.5	124,800	34.969	34.991
37	40	1.5	123,600	35.258	35.588
30	33	1.5	123,600	35.306	35.705

## Nomenclature

$h_{\mathrm{in}}$	the internal surface coefficient of heat transfer (8.7 W·m^−2^·°C^−1^)
$h_{\mathrm{out}}$	the external surface coefficient of heat transfer (19 W·m^−2^·°C^−1^)
$\lambda_{B}$	the thermal conductivity of the blocks (W·m^−1^·°C^−1^)
$\lambda_{\mathrm{MJ}}$	the thermal conductivity of masonry joints (W·m^−1^·°C^−1^)
L	the length of the wall (mm)
H	the height of the wall (mm)
W	the width of the wall (mm)
d	the width of masonry joints (mm)
$T_{\mathrm{out}}$	the temperature boundary condition of the outer surface of the wall (°C)
$T_{\mathrm{in}}$	the temperature boundary condition of the inner surface of the wall (°C)
$Q_{\mathrm{AHF}}$	the average heat transfer of a single grid integral point under steady state of the wall (W)
$n_{W}$	the number of grids in the direction of the thickness extension paths of the wall (1)
$n_{H}$	the number of grids in the direction of the height extension paths of the wall (1)
ΔT	temperature range between the inner and outer surface of the wall under steady state (°C)
Q	the total heat flux from the outer wall to the inner wall in a single period after the transient heat transfer temperature waveform changes stably (J·m^−2^)
$Q^{\prime }$	the latent heat flux from the outer wall to the inner wall in a single period after the transient heat transfer temperature waveform changes stably (J·m^−2^)
K	the theoretical heat transfer coefficient of the wall under steady state (W·m^−2^·°C^−1^)
R	the heat transfer resistance of the wall under steady state (W^−1^·m^2^·°C)
$K_{d}$	the numerical heat transfer coefficient of the wall under steady state (W·m^−2^·°C^−1^)
$K_{m}$	the numerical heat transfer coefficient of the model under steady state (W·m^−2^·°C^−1^)
${K_{m}}^{\prime }$	the corrected numerical heat transfer coefficient of the model under steady state (W·m^−2^·°C^−1^)
Δt	the calculating effective time period (s)
